# Improvement of the Culture Medium for the Dichlorvos-Ammonia (DV-AM) Method to Selectively Detect Aflatoxigenic Fungi in Soil

**DOI:** 10.3390/toxins10120519

**Published:** 2018-12-05

**Authors:** Kimiko Yabe, Haruna Ozaki, Takuya Maruyama, Keisuke Hayashi, Yuki Matto, Marika Ishizaka, Takeru Makita, Syun-ya Noma, Kousuke Fujiwara, Masayo Kushiro

**Affiliations:** 1 Department of Environmental and Food Sciences, Faculty of Environmental and Information Sciences, Fukui University of Technology, 3-6-1 Gakuen, Fukui-shi, Fukui 910-8505, Japan; campanula.gysopila3357@gmail.com (H.O.); takuya12fukui1katsudon5@ezweb.ne.jp (T.M.); ki-hayashi115@docomo.ne.jp (K.H.); yuuki-mouse0220@docomo.ne.jp (Y.M.); marika19951212@yahoo.co.jp (M.I.); fklih_tkr041@docomo.ne.jp (T.M.); shunya.futp@outlook.com (S.-y.N.); radrill8968@gmail.com (K.F.); 2 Food Research Institute, National Agriculture and Food Research Organization (NARO), 2-1-12 Kannon-dai, Tsukuba-shi, Ibaraki 305-8642, Japan; kushirom@affrc.go.jp

**Keywords:** aflatoxins, *Aspergillus nomius*, *Aspergillus parasiticus*, culture medium, dichlorvos-ammonia (DV-AM) method, deoxycholate, detergent, *Rhizopus* sp.

## Abstract

The dichlorvos-ammonia (DV-AM) method is a simple but sensitive visual method for detecting aflatoxigenic fungi. Here we sought to develop a selective medium that is appropriate for the growth of aflatoxigenic fungi among soil mycoflora. We examined the effects of different concentrations of carbon sources (sucrose and glucose) and detergents (deoxycholate (DOC), Triton X-100, and Tween 80) on microorganisms in soils, using agar medium supplemented with chloramphenicol. The results demonstrated that 5–10% sucrose concentrations and 0.1–0.15% DOC concentrations were appropriate for the selective detection of aflatoxigenic fungi in soil. We also identified the optimal constituents of the medium on which the normal rapid growth of *Rhizopus* sp. was completely inhibited. By using the new medium along with the DV-AM method, we succeeded in the isolation of aflatoxigenic fungi from non-agricultural fields in Fukui city, Japan. The fungi were identified as *Aspergillus nomius* based on their calmodulin gene sequences. These results indicate that the new medium will be useful in practice for the detection of aflatoxigenic fungi in soil samples including those from non-agricultural environments.

## 1. Introduction

Aflatoxins (AFs) are highly toxic, carcinogenic, and teratogenic secondary metabolites produced by fungi [1]. The contamination of agricultural commodities with AFs is not only a serious health hazard to humans and animals, causing hepatotoxicity as an acute toxicity or hepatic cancer as a chronic toxicity [2] but also a cause of huge economic losses worldwide.

AFs are produced mainly by certain strains of *Aspergillus flavus* Link and *Aspergillus parasiticus* Speare [1]. These fungi have been thought to be distributed mostly in tropical and subtropical regions [3,4,5]. Aflatoxigenic fungi are present in soil as conidia, sclerotia, or hyphae, which act as primary inoculants for directly infecting peanuts or for infecting aerial crops such as maize, sugarcane, and cotton [6]. These fungi have also been reported in temperate regions (especially when droughts have occurred) in the United States and other countries [3].

Several other species of the *Aspergillus* group have been reported to produce AFs: *A. nomius* [7,8,9], *A. pseudotamarii* [10,11], *A. bombycis* [12], *A. parvisclerotigenus* [13,14,15], *A. minisclerotigenes* [13], and more, most of which are reported to occur in temperate regions. Additional unknown aflatoxigenic species may be present, and they may be widely distributed in the world. The clarification of the distribution as well as the habitats of aflatoxigenic fungi is important to effectively prevent aflatoxin contamination in crops, feed, and food.

Many researchers have reported the isolation of aflatoxigenic fungi from the soil in agricultural fields (e.g., cotton, peanut and maize fields), and the researchers usually used multiple procedures: (1) the isolation of *Aspergillus* section Flavi using *Aspergillus flavus* and *parasiticus* agar (AFPA) medium [16], modified rose Bengal agar (MRBA) medium [17], or coconut agar medium [18]; (2) the confirmation of aflatoxin production by the isolated strains with the use of thin layer chromatography (TLC), high-performance liquid chromatography (HPLC) [19,20,21,22], an enzyme-linked immunosorbent assay (ELISA) [23,24,25,26,27,28], and other methods; (3) the identification of aflatoxigenic fungi using a polymerase chain reaction (PCR) or a real-time PCR [29,30,31], and other methods. Since methods (1) and (3) are useful for discriminating fungi belonging to *Aspergillus* section Flavi, the ability of the fungi to produce aflatoxins need to be confirmed by another analytical method noted in method (2). These methods are useful and fruitful techniques but are time-consuming and often require some special facilities, especially in step (2). For more practical use, a single-step method for the selective detection of aflatoxigenic fungi from environmental samples is desirable.

**Figure 1:**
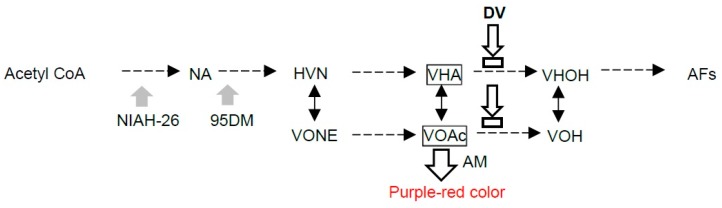
Outline of the DV-AM method. DV inhibits the versiconal hemiacetal acetate (VHA) esterase in AF biosynthesis to cause an accumulation of VHA and versiconol acetate (VOAc) instead of AFs in the mycelia. AM vapor treatment change the colors of these substances from pale yellow to brilliant purple-red. Mutation sites of *A. parasiticus* NIAH-26 and 95DM strains are also indicated (gray thick arrows). AFs: aflatoxins, AM: ammonium vaper, NA: norsolorinic acid, VHOH: versiconal, VOH: versiconol.

We previously developed a sensitive and simple visual detection method, the dichlorvos-ammonia (DV-AM) method [32], in which fungi are first cultured on AF-inducible agar medium supplemented with dichlorvos (DV) for 2–6 days, and the resulting colonies are then treated by ammonia (AM) vapor to monitor the mycelial color changes from pale yellow to brilliant purple-red. The color change of the colony is due to color changes of aflatoxin precursors, i.e., versiconal hemiacetal acetate (VHA) and versiconol acetate (VOAc), which accumulate in the mycelia by the inhibition of an AF biosynthetic enzyme, VHA esterase, by DV [33] (Figure 1A).

Since these precursors contain anthraquinone moieties in their structures, the mycelial colors are markedly changed from the original yellowish colors to purple-red through the pH change by ammonia vapor. Monitoring this color change makes it easy to identify aflatoxigenic fungi, as the colors of non-aflatoxigenic fungi do not change. We demonstrated that with the DV-AM method, aflatoxigenic fungi were isolated from sorghum fields in Japan [34], and that this method was also useful for the isolation of aflatoxigenic as well as atoxigenic fungi from soils of a maize field in Mexico [35].

In our previous work, we routinely used GYD (2% glucose, 0.5% yeast extract and 0.05% deoxycholate (DOC)) medium for the screening of aflatoxigenic fungi [34,35], and DOC was added to this medium to make the fungal colonies compact. We occasionally observed that the red color intensities of the colonies were relatively too weak to accurately detect the target fungi in this medium [35,36]. In addition, at an early stage of this work, we noted that no red colonies of aflatoxigenic fungi were detected when conidiospores of aflatoxigenic fungi were mixed with soils from the Fukui University of Technology (FUT) campus and then analyzed by the DV-AM method in GY(2-0.5) (2% glucose, 0.5% yeast extract) medium.

In the present study, we examined the effects of carbon sources and detergents as well as their concentrations to optimize media for the DV-AM method. We were able to establish ‘recipes’ of the optimal medium constituents for aflatoxigenic fungi as well as *Aspergillus* section Flavi. Using this medium, we isolated aflatoxigenic fungi from a non-agricultural field in Fukui city, Japan. This is the first report that aflatoxigenic fungi are present in the Hokuriku area in Japan. We will report the distribution of aflatoxigenic fungi in Japan in another paper.

## 2. Results

### 2.1. The Effect of the Carbon Source on the DV-AM Method’s Results

**Figure 2:**
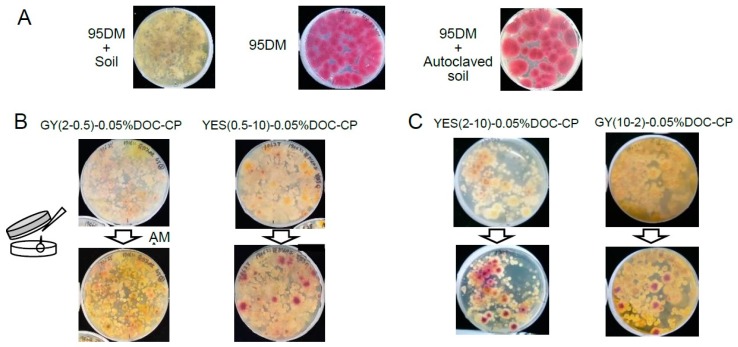
DV-AM method using various culture media. (**A**) Effect of soil (approx. 15 mg) on the growth of 95DM fungi on GY(2-0.5) agar medium. Conidiospores of the 95DM mutant were mixed with (left) or without (center) the soil suspension and then inoculated onto GY agar medium and cultured at 28 °C for 3 days. The same conidiospores were also mixed with the autoclaved soil and cultured (right). The resulting colonies were treated with AM solution and then observed from the back side of the plate. (**B**) The soil suspension of the FUT flowerbed soil, which endogenously contained aflatoxigenic fungi, was inoculated onto GY(2-0.5)-0.05% DOC-CP-DV (left) or YES(0.5-10)-0.05% DOC-CP-DV (right) medium and then cultured. The plates were treated with AM. (**C**) The same soil suspension was inoculated onto YES(10-2)-0.05% DOC-CP-DV (left) and GY(10-2)-0.05% DOC-CP-DV (right) medium and then cultured. The plates were treated with AM.

Since the *A. parasiticus* strain 95DM accumulates norsolorinic acid (NA), which is an AF precursor containing anthraquinone moiety in its molecule [37], the colonies of 95DM showed a purple-red color following AM treatment on GY(2-0.5) plates without DV (Figure 2A, left). In contrast, when 95DM was inoculated with the FUT campus soil and cultured, no red colonies were detected by AM treatment (Figure 2A, middle). No red colonies were observed even when a tenfold-diluted soil suspension was used (data not shown). In contrast, when the autoclaved soil was used in the same experiment, only red colonies appeared (Figure 2A, right). These results indicated that the growth of other microorganisms in the soil completely inhibited the growth of the 95DM strain.

During this study, we discovered that a FUT flowerbed soil endogenously contained some aflatoxigenic fungi. To examine the competency of media for the sensitive detection of aflatoxigenic fungi, the same FUT soil (approx. 30 mg) was cultured on GY(2-0.5)-0.05% DOC-CP-DV or YES(0.5-10)-0.05% DOC-CP-DV medium (Figure 2B) to which an antibiotic, chloramphenicol (CP) was added. AM treatment caused the appearance of some small pink colonies together with those of other microorganisms in the former medium. In contrast, brilliant red colonies appeared in the YES(0.5-10)-0.05% DOC-CP-DV medium, suggesting that a higher concentration of the carbon source is appropriate for the sensitive detection of aflatoxigenic fungi.

To determine which carbon source (sucrose or glucose) is appropriate for the DV-AM method, we analyzed the FUT soil using either YES(2-10)-0.05% DOC-CP (Figure 2C, left) or GY(10-2)-0.05% DOC-CP medium (Figure 2C, right). Although the colonies of aflatoxigenic fungi in the soil were brilliant red on both plates, the GY(10-2)-0.05% DOC-CP medium plate became remarkably dark due to the Mallard reaction through autoclaving (Figure 2C, right). A high concentration of sucrose seems to be more appropriate than glucose when autoclaving is used for pasteurization of the medium.

**Figure 3:**
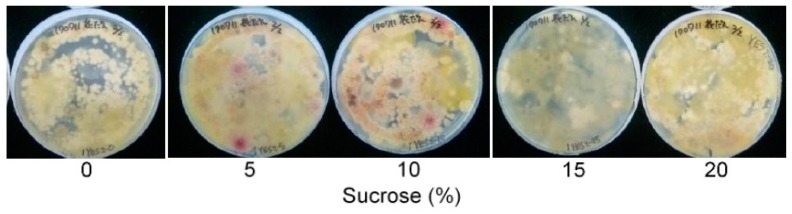
The FUT soil suspension was cultured on the 2% yeast extract-0.05% DOC-CP-DV agar medium supplemented with the specified concentration of sucrose. After AM treatment, the plates were observed.

To determine the appropriate concentration of the carbon source, we examined the same soil using the same medium except for the varied concentration (0–20%) of sucrose (Figure 3). A sucrose concentration from 5% to 10% was appropriate for the detection of aflatoxigenic fungi, and both lower as well as higher concentrations of sucrose did not show any red colonies. We thus decided that a 10% sucrose concentration would be useful for the DV-AM method.

### 2.2. The Effect of Detergents in the Media

**Figure 4:**
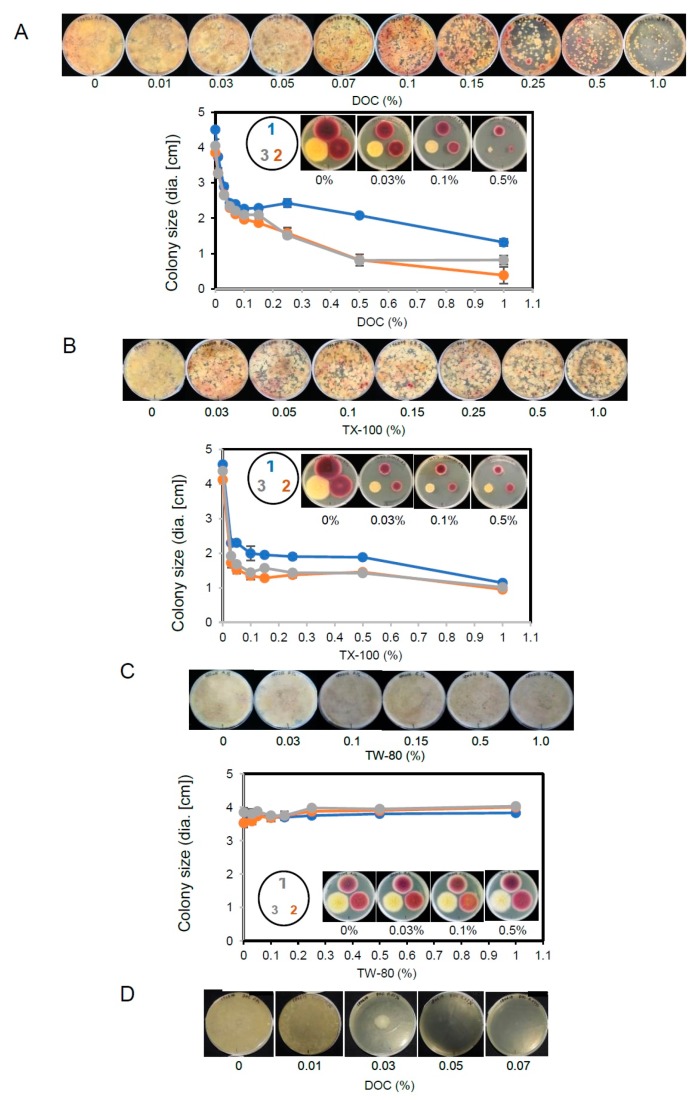
The effect of detergents on the growth of aflatoxigenic fungi and other microorganisms. (**A**) Soil suspension of FUT (approx. 30 mg of soil) was cultured on YES(2-10)-CP-DV medium supplemented with various concentrations of DOC for 3 days, and the resulting plate was treated with AM vapor (upper). An aflatoxigenic FUT-A1 strain (1, blue line), the aflatoxigenic *A. parasiticus* SYS-4 strain (2, orange line), and the non-aflatoxigenic *A. parasiticus* NIAH-26 strain (3, gray line) were cultured on the same plates as in the above-described experiments for 3 days. After AM treatment, the diameter of each colony was measured. (**B**) The same experiments as in panel (**A**) were done using TX-100 instead of DOC. (**C**) The same experiments as in panel (**A**) were performed using TW-80 instead of DOC. (**D**) A *Rhizopus* sp. strain was inoculated onto the center of the same plate as in panel (**A**) and cultured for 3 days.

We examined the effect of detergents (DOC, Triton X-100 (TX-100), and Tween 80 (TW-80)) on the growth of aflatoxigenic fungi and other microorganisms in FUT soil (Figure 4). When the FUT soil was analyzed using YES-CP-DV medium supplemented with various concentrations of DOC (Figure 4A, upper), colonies of the aflatoxigenic fungi were not detected up to 0.03% DOC, which is a concentration at which other microorganisms grew dominantly. The aflatoxigenic red colonies appeared above 0.1% DOC, and the higher the concentration of DOC, the lower the number of other microorganisms and the clearer the aflatoxigenic fungi were. These results suggested that aflatoxigenic fungi are more tolerant to detergent than other microorganisms.

When three strains, i.e., FUT-A1 (an isolate from a FUT flowerbed soil), *A. parasiticus* SYS-4, and *A. parasiticus* NIAH-26 were cultured on the same plates containing various DOC concentrations for 3 days, each fungal size changed with the increase in the DOC concentration (Figure 4A, lower). The diameter of each colony became approx. 50% smaller at around 0.1% DOC, and the concentrations above 0.5% resulted in a marked decrease in the size of the two *A. parasiticus* strains, the colonies of which had been difficult to detect visually. Although we were able to determine that the FUT-A1 strain belongs to *A. nomius* in this investigation, the FUT-A1 colony was significantly larger than the *A. parasiticus* colonies at higher DOC concentrations (0.25–1.0%). These results indicated that *A. nomius* is more tolerant to DOC than *A. parasiticus*, and that a DOC concentration >0.15% may be more appropriate for the detection of the *A. nomius* strain.

We performed the same experiments using TX-100, which has also been used to make colonies compact [35]. At 0.03% TX-100, the sizes of the aflatoxigenic fungi and other microorganisms drastically decreased, and their sizes did not significantly change at concentrations up to 1.0% (Figure 4B, upper panel). When three strains were cultured on the same plates (Figure 4B, lower), the sizes of all colonies decreased to approx. 35% of the original sizes observed at 0.03% TX-100. Their sizes did not change markedly with the increase in the TX-100 concentration, whereas FUT-A1 appeared slightly more tolerant to TX-100 compared to the other strains.

When the same experiments were done using TW-80, no aflatoxigenic fungi were detected at all concentrations up to 1% TW-80 (Figure 4C, upper panel). None of the concentrations of TW-80 used affected the growth of the three strains (Figure 4C, lower). These results indicated that TW-80 is not useful for making a selection medium for aflatoxigenic fungi.

Contamination by *Rhizopus* sp. fungi usually disturbs the observation of other microorganisms in an agar plate, because *Rhizopus* sp. grows rapidly and spreads throughout the petri dish surface within 1 day. When we inoculated a *Rhizopus* sp. strain onto the center of agar plates containing various concentrations of DOC, the mycelial density significantly decreased at 0.01% DOC, and fungal growth was greatly inhibited at 0.03% DOC. DOC concentrations >0.05% completely inhibited the growth of *Rhizopus* sp. (Figure 4D). Similar strong inhibition was also observed when 0.03% TX-100 was used, whereas the small colonies (approx. 5 mm dia.) remained at 1.0% TX-100 (data not shown) [38].

These results indicated that among the three detergents tested, DOC is the most appropriate detergent to selectively detect aflatoxigenic fungi by the DV-AM method. Although the *A. nomius* strain was more tolerant to DOC than the other aflatoxigenic strains, the addition of 0.1% DOC to the medium was appropriate for the detection of all aflatoxigenic strains by the DV-AM method. This concentration was also shown to be useful to completely inhibit the growth of *Rhizopus* sp. fungi.

### 2.3. The Characterization of Aflatoxigenic Fungi in Soils and the Recovery of the Aflatoxigenic Fungi from Soils

Since we found that soil from a flowerbed on the FUT campus contained aflatoxigenic fungi, we investigated the conditions of the fungi in soils and the recovery rates of the aflatoxigenic fungi from the soils for the practical use of the DV-AM method.

**Figure 5:**
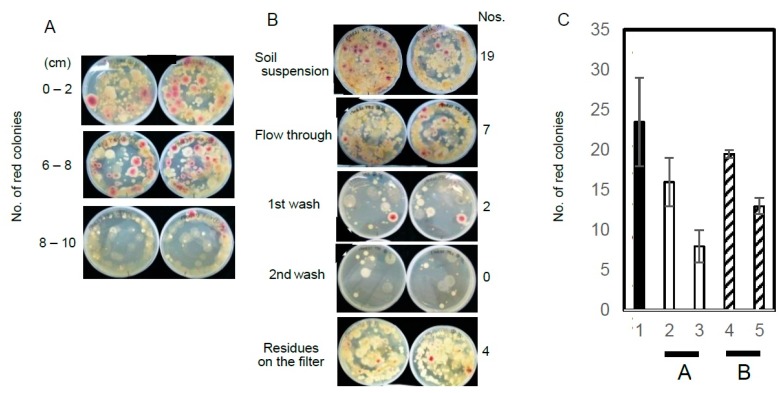
The collection and storage conditions of the soils. (**A**) Soil samples from the FUT flowerbed containing aflatoxigenic fungi were respectively collected from each depth (surface to 2 cm depth, 2–4 cm, 4–6 cm, 6–8 cm, and 8–10 cm) and inoculated and cultured on YES(2-10)-0.05% DOC-CP-DVmedium. After treatment with AM vapor, red colonies were observed. (**B**) A suspension of the FUT soil containing the aflatoxigenic fungi was filtrated through 50 μm mesh, and the filtrate was then pooled. The filter was then washed twice with the same volume of 0.05% TW-80 aqueous solution, and each filtrate was pooled. The 1st and 2nd filtrates were respectively analyzed by the DV-AM method. The residue on the filter was also analyzed by suspending it with the same volume of the TW-80 aqueous solution (data not shown). The aflatoxigenic fungi in the resulting samples were examined by the DV-AM method. The numbers of red colonies after AM treatment are shown. (**C**) The effect of soil addition on the growth of the aflatoxigenic *A. parasiticus* SYS-4 strain on agar plates. The SYS-4 conidiospores were inoculated together with different concentrations of soil A (open bar) or soil B (striped bar), both of which had been confirmed not to contain aflatoxigenic fungi, or without soil (black bar). The numbers of red colonies on each plate after AM treatment were counted.

To determine the soil depths that are appropriate for the collection of aflatoxigenic fungi, soil samples were collected at 2 cm deep intervals from the surface to 10 cm depth and then subjected to the DV-AM method (Figure 5A). Significant numbers of aflatoxigenic fungi were obtained ranging from the surface to 8 cm depth, but were hardly detected below 8 cm; the same was true of other microorganisms.

To determine the status of the aflatoxigenic fungi in the soil, we filtrated the soil suspensions through 30 μm mesh. Most of the fungal colonies were detected in the flow-through, first wash, and second wash samples (Figure 5B). These results suggested that most of the aflatoxigenic fungi were present as conidiospores.

To investigate the recovery of the aflatoxigenic fungi from soils by this method, we applied the DV-AM method to determine recovery of SYS-4 strains, which had been mixed with different concentrations of FUT soil suspensions. We used two types of soil subsamples (which we named ‘A’ and ‘B’) in which no aflatoxigenic fungi had been detected. When 15 mg of soil A and 15 mg of soil B was added to SYS-4 spores and analyzed by the DV-AM method, approx. 68% and 83% of aflatoxigenic fungi were recovered from soil A and soil B, respectively. When twice that amount of either soil was used, the recovery of the aflatoxigenic fungi decreased: ≥34% of aflatoxigenic fungi could be recovered (Figure 5C). Similar results were obtained when strain 95DM conidiospores were used instead of SYS-4 conidiospores (data not shown). These results suggest that quantitative measurements of fungi in soils require an excessive dilution of the soils or another quantitative method, although the results would be dependent on each soil.

For our evaluation of the optimal soil storage conditions, an aliquot of the same FUT soil was treated by freeze-thawing. One to three times freeze-thawing did not significantly affect the viabilities of the fungi, and the results obtained with the soils and the soil suspensions were almost the same (data not shown).

### 2.4. Application of the Improved Medium for the DV-AM Method

**Figure 6:**
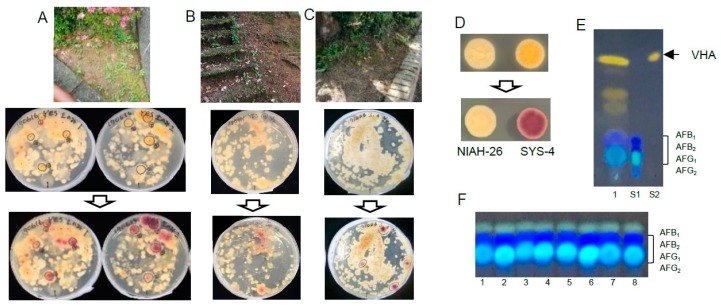
Application of the DV-AM method using the YES(2-10)-0.1% DOC-CP medium for the isolation of aflatoxigenic fungi from soils (approx. 30 mg each) in Fukui city. (**A**) A flowerbed at the FUT campus; (**B**) a mountain road; (**C**) a park. The soils were collected and analyzed by the DV-AM method. Colonies were observed before and after AM treatment. Some colonies that were expected to be aflatoxigenic fungi are lined with black circles. (**D**) The morphology of the colonies of aflatoxigenic fungi on the resulting plate. Colonies of *A. parasiticus* NIAH-26 (left) and SYS-4 (right) were observed from the plate’s underside before (upper) or after (lower) AM treatment. (**E**) A part of a colony of FUT-A1 on a YES(2-10)-0.05% DOC-CP-DV plate together with the agar underneath were picked up and extracted with ethyl acetate. The resulting extract was analyzed by TLC (lane 1) together with standards of aflatoxin mix (S1) and VHA (S2). (**F**) Parts of purified red colonies on GY(2-0.5) plate together with the agar underneath were picked up and extracted with ethyl acetate, respectively. The resulting extracts were analyzed by TLC (lanes 1–8).

We attempted to isolate aflatoxigenic fungi from three sites in Fukui city using the YES(2-10)-0.1% DOC-CP medium and the DV-AM method. The soil from the flowerbed, which was from the same site from which FUT-A1 was isolated, showed brilliant red colonies (Figure 6A). Soils from a mountain road (Figure 6B) and a city park (Figure 6C) in Fukui city also showed some red colonies on the plates. The genus *Aspergillus* including aflatoxigenic fungi appeared on this agar medium as colonies with white fluffs from the surface; in contrast, flat yellow colonies showing radiation-like wrinkles were observed underneath the plates (Figure 6D). Based on these morphological features, we could expect colonies of the aflatoxigenic fungi.

When an aliquot of a FUT-A1 colony on YES(2-10)-0.1% DOC-CP agar medium together with the agar part underneath the colony was extracted and analyzed by TLC, VHA and aflatoxins (mainly AFB_1_ and AFG_1_) were observed (Figure 6E). When a similar experiment using some colonies on GY(2-0.5) plates after purification was conducted, mainly AFB_1_ and AFG_1_ were observed (Figure 6F). These results indicated that the fungi were B-group and G-group AF producers.

The aflatoxigenic fungi consisted of macro nucleate and colorless conidiophores producing monophialide conidiogenous cells on the swollen, spherical apex, which are characteristic features of *Aspergillus* isolates [39]. The colonies of aflatoxigenic fungi were diffuse and light green (data not shown). DNA sequencing of the calmodulin gene of the FUT-A1 followed by a BLAST analysis demonstrated that the aflatoxigenic fungi obtained were *A. nomius*, based on their *cmd* gene sequences.

These results indicated that the culture medium developed herein is practically useful for the detection of aflatoxigenic fungi in various soils.

## 3. Discussion

### 3.1. The Selection Medium for Aflatoxigenic Fungi

Soils generally contain various kinds of microorganisms, and some of them inhibit growth of aflatoxigenic fungi. Although CP was added to the medium to inhibit bacteriological growth in this work, addition of soil to spores of aflatoxigenic fungi decreased the recovery rate of aflatoxigenic fungi (Figure 2A and Figure 5C), suggesting that eukaryotic microorganisms such as yeasts and other fungi inhibit growth of aflatoxigenic fungi in the agar plates. Hua et al. reported that some saprophytic yeasts inhibited growth of aflatoxigenic fungi as well as aflatoxin production [40]. Munoz reported that *Saccharomyces cerevisiae* inhibited growth of *A. nomius* VSC 23 strains [41], whereas we did not herein investigate other microorganisms except aflatoxigenic fungi in detail. Many researchers have searched for microorganisms inhibitory to aflatoxin production or growth of aflatoxigenic fungi to prevent aflatoxin contamination in feed and food. We also isolated many microorganisms for this purpose, whereas inhibitory activity of each of them had been commonly weak for practical use (data not shown). This work might show that combination of some microorganisms as well as their mass might to be effective to effectively inhibit aflatoxin production or growth of aflatoxigenic fungi.

This work indicated that the YES(2-10)-0.1% DOC-CP medium is appropriate for selective detection of aflatoxigenic fungi in soils. High concentration (5–10%) of sucrose was appropriate to make brilliant purple-red colonies among many colonies of other microorganisms on the medium. Also, since *Aspergillus* section Flavi including aflatoxigenic fungi was found to be more tolerant to DOC than other microorganisms, addition of the appropriate concentration (0.1%) of DOC to the medium was useful to more selectively detect the aflatoxigenic fungi than those of other microorganisms. Also, colonies of *Aspergillus* section Fravi fungi including aflatoxigenic fungi showed characteristic morphology: Flat colonies having radiation-like wrinkles from the underside. Confirmation of the morphological characters and the color change from pale yellow to purple red by AM treatment can provide more promising determination of aflatoxigenic fungi.

TX-100 has also been commonly used to make fungal colonies compact, whereas concentration dependency of TX-100 on mycelial sizes were different from that of DOC. Since low concentration of TX-100 drastically decreased the sizes of aflatoxigenic fungi as well as other microorganisms, discrimination of the aflatoxigenic fungi from others were not easy. Interestingly, TW-80 did not affect the growth of the aflatoxigenic fungi as well as other microorganisms including fungi belonging to genus *Rhizopus*.

The mechanisms of growth inhibition by detergents are not known. It is generally accepted that a detergent molecule or micelle binds to cell membranes of microorganisms and it affect certain functions of cells [42]. For examples, adsorption of a detergent to cell membrane to cause cell disruption. Also, it might cause inactivation of membrane (intrinsic or surface) enzymes. It might cause denaturation of some proteins essential for growth of the microorganisms. The effect of either detergent might be induced by effects or inactivation of some essential fungal enzymes. We will continue to search for different detergents useful for making a better selection medium for the aflatoxigenic fungi.

This work also showed that *A. nomius* strain was more tolerant than *A. parasiticus*, and we also found that *A. flavus* showed similar tolerance to DOC to *A. parasiticus*. Since Massi et al. reported primer sequences of PCR analysis for selective detection of *A. nomius* strains [43], combination of the DV-AM method using YES(2-10)-0.15% DOC-CP agar medium and PCR analysis using the primers will make it possible to identify *A. nomius* strains from environmental samples such as soils.

In this work, we did not investigate other microorganisms in soils, which were *Rhizopus* sp. and expected to be yeasts, *Penicillium*, and so on. We often observed some pale purple colonies on the selection medium and their colors changed to more intense purple-red by AM treatment. These colonies were smaller than those of aflatoxigenic fungi and we confirmed that these fungi did not produce any aflatoxins by TLC analyses (data not shown). These color metabolites affected with AM treatment seems to be important to study soil microorganisms.

This work thus showed that microorganisms in soils inhibited growth of the aflatoxigenic fungi. Although 10-fold dilution of soil did not affect the inhibition, combination of several microorganisms may be useful for inhibition of the fungal growth in crops. Soils might be able to regulate infection of aflatoxigenic fungi in crops.

This work clearly showed that most of all the aflatoxigenic fungi were present as condiospores in the soils. Also, the soils were obtained from non-agricultural places such as FUT campus and a city park. Since crops heavily infected with the aflatoxigenic fungi has not been reported in Japan except at Miyazaki University, the reason the conidiospores are present in the non-agricultural fields remains to be studied. We found that the aflatoxigenic fungi were present at up to 8 cm depth from the surface of the soil. Horn et al. reported that conidia of *A. flavus* and *A. parasiticus* do not readily move downward beyond the upper 6 cm of soil surface, when those conidia were applied to the soil surface [6]. In contrast, the fungi were present in cultivated fields up to 30 cm in depth [6,44], probably due to the mixing of soil during plowing or to colonization of organic matter at such depth. Since the flowerbed in the FUT was not been plowed for long time, conidiospores are likely conveyed from another place and dropped onto the non-agricultural field and then accumulated in soils. The detailed distribution mechanism remains to be studied.

### 3.2. Application of the Selection Medium with the DV-AM Method for Aflatoxigenic Fungi in Non-Agricultural Fields in Fukui City

Aflatoxigenic fungi have been thought to be rare in Japan, especially in the area of the north of the central part in Japan [45]. However, this work using the DV-AM method and the resulting improved medium demonstrated that soils from some area in Fukui city contained the aflatoxigenic fungi. The aflatoxigenic fungus FUT-A1 from soil of a FUT flowerbed was identified to be *A. nomius*. Although the aflatoxigenic fungi have been isolated from agricultural fields in Okinawa area [36], Kyusyu area [46], Kanto area [47,48], and Ibaraki prefecture [34,48,49]. This is the first report that aflatoxigenic fungi are present in Hokuriku area (the Japan Sea side area with a heavy snow in winter), where aflatoxigenic fungi had not been expected meteorologically. Also, we obtained them from non-agricultural fields, which produced interesting results because aflatoxigenic fungi have been detected mainly in agricultural fields in the USA and in other countries.

These results suggested that the DV-AM method using the improved medium will be a promising method for clarification of distribution and movement of the fungi in various environments. In fact, we have already started screening of aflatoxigenic fungi in many places and various environments in Japan. We will report those results in another paper soon.

## 4. Conclusions

In this study, we demonstrated that addition of appropriate concentrations of DOC and sucrose to agar medium are useful for selective detection of aflatoxigenic fungi using the DV-AM method. Using this semi-selective medium and DV-AM method, some aflatoxigenic fungi were successfully isolated from some non-agricultural field in Fukui city, Japan. These conditions will be useful for screening of aflatoxigenic fungi from soils from various environments.

## 5. Materials and Methods

### 5.1. Microorganisms

*A. parasiticus* SYS-4 (NRRL2999) was used as an aflatoxigenic strain. *A. parasiticus* 95DM is a norsolorinic acid-accumulating mutant strain of the SYS-4 strain. *A. parasiticus* NIAH-26 is a mutant strain of the SYS-4, which produces neither AFs nor any pigmented AF precursors [50]. An aflatoxigenic fungi was herein isolated from the soil in Fukui University of Technology, and named as FUT-A1, which was identified as *A. nomius* based on its calmodulin DNA sequence. Aflatoxigenic fungi isolated from soils in Fukui city were named using the prefix FUT-. FUT-A1 strain among them was used in this work. *Rhizopus* sp. was isolated from a soil in FUT, which was identified based on its morphological characters [51].

### 5.2. Media

For the description of each medium, we named the concentrations of yeast extract and the carbon sources used by using a hyphen after the abbreviation of the medium. When necessary, a blanket together with the hyphen is also used to avoid confusion. We routinely used YES(2-10)-0.1% (*w*/*v*) DOC-CP agar medium (2% yeast extract (Nacalai Tesque, Kyoto, Japan), 10% sucrose (Nacalai Tesque), 0.1% Na DOC (Fujifilm Wako Pure Chemical Co., Osaka, Japan), 0.1 g CP (chloramphenicol (Nacalai Tesque))/L, and 2% agar (Nacalai Tesque)) for the DV-AM method. The medium YES(2-10)-0.15% DOC-CP or YES(2-10)-0.05% DOC-CP medium was also used when specified.

To examine the effect of carbon sources, we used the 2% yeast extract-0.05% DOC-CP medium supplemented with various concentrations of sucrose.

To examine the effect of the detergent on the growth of microorganisms on agar plates, we used YES(2-10)-CP agar medium supplemented with various concentrations of DOC, TX-100 (polyethylene glycol mono-p-isooctylphenyl ether (Nacalai Tesque)), or TW-80 (polyoxyethylene sorbitan monooleate (Nacalai Tesque)). Potato dextrose agar medium (PDA (BD Difco, Detroit, MI, USA)) was used for morphological characterization. The GY(2-0.5) agar medium (2% glucose (Nacalai Tesque), 0.5% yeast extract, 2% agar) was used for the purification of each fungus.

For the screening of the aflatoxigenic fungi from soils, 20 μL of 100-fold diluted DV (dichlorvos Fujifilm Wako)) methanol solution was spread onto YES(2-10)-0.1% DOC-CP agar medium to make YES(2-10)-0.1% DOC-CP-DV and then stored at 4 °C until usage.

### 5.3. DV-AM Method

Each soil sample (approx. 0.15 g) was suspended in 0.5 mL of 0.05% TW-80 aqueous solution, and the resulting suspension (50 μL or 100 μL) was then spread onto YES-0.1% DOC-CP-DV agar plates or other plates. The plates were put upside-down and then cultured at 28 °C in darkness for 3 days. Each plate was treated with 200 μL of ammonium hydroxide solution (28%, reagent grade, Wako, Osaka, Japan) put in the inside of the lid of the petri plate. When color changes of colonies from pale yellow to red were observed from the underside of the plates, conidiospores of each red colony (candidate aflatoxigenic fungi) were immediately picked up with a sterile toothpick and inoculated onto a new GY(2-0.5) plate and cultured. When *Rhizopus* sp. was observed on the same plate, YES(2-10)-0.1% DOC-CP-DV medium was used for the second purification of the isolated fungus. The resulting fungal isolates were further purified by three repetitions of single conidiospore isolation.

For the improvement of the culture media, a soil suspension (approx. 15 mg) of the FUT flowerbed was inoculated onto a YES(10-2)-CP-DV agar plate supplemented with various concentrations of DOC, TX-100, and TW-80 and then cultured at 28 °C for 3 days. Each conidiospore suspension (2 μL each) of the FUT-A1, SYS-4, and NIAH-26 strains was inoculated onto the same plate and then cultured for 3 days. The resulting plates were then treated with AM vapor, and color changes were then observed from the back side of the plate. The diameters of the resulting colonies were respectively measured.

After the inoculation of fungi or soils, all petri dishes were shielded with Silkylite tape (Tokyo Eizai Laboratory, Tokyo, Japan) and then cultured.

### 5.4. Collection and Storage of Soils

Soil samples (approx. 10g) present up to 5 cm in depth were routinely collected into plastic bags from three sites in Fukui city, Japan: a flowerbed on the FUT campus, a mountain road, and a park in August–October 2017, and were kept at 4 °C until use.

For the determination of the appropriate depth for soil collection, soils in the flowerbed on the FUT campus were collected from each plate at five 2 cm depths from the surface.

Regarding the storage conditions of the soils, we repeated three sessions of freezing at 80 °C followed by thawing at room temperature. The resulting soil at each step was analyzed by the DV-AM method.

### 5.5. The Morphology of the Aflatoxigenic Fungi in FUT Soil

For the analysis of the morphology (conidiospores, sclerotia, or mycelia) of aflatoxigenic fungi in the FUT flowerbed soil, a soil suspension with 0.05% TW-80 aqueous solution from a FUT flowerbed was filtered through a 50 μm mesh sheet, and the flow sample was pooled. The residue on the filter was washed twice with the same volume of the solution (1st wash and 2nd wash). The residue remaining on the filter was suspended with the same volume of the solution. Each pooled fraction was inoculated onto a YES-0.1% DOC-CP-DV plate and analyzed by the DV-AM method.

### 5.6. Recovery of Aflatoxigenic Fungi from Soils by the DV-AM Method

A SYS-4 spore suspension was mixed with a soil suspension containing 15 mg or 30 mg FUT soil, in which no aflatoxigenic fungi had been detected, in a total volume of 260 μL. The resulting solution (100 μL each) was respectively inoculated onto YES-0.1% DOC-CP-DV medium and then cultured for 3 days. After AM treatment, the number of SYS-4 red colonies on the plate was counted. The recovery rate of the colonies was calculated by comparison with those (approx. 50 conidiospores) in the absence of soil. The same experiment was done in duplicate using two different FUT soils.

### 5.7. Characterization of the Isolated Fungi

To confirm the AF productivity of the FUT-A1 strain and other fungi isolated from soils of Fukui city, Japan, we cut out an aliquot of the fungal colony together with the agar underneath the colony on an agar plate and transferred it to a new 1.5 mL microtube. Metabolites in the samples were extracted with 0.3 mL of ethyl acetate followed by mixing with a vortex mixer. After centrifugation at 10,000× *g* for 2 min, the upper extract (10 μL) was analyzed by TLC using a silica gel plate (silica gel 60, #5721; Merck, Rahway, NJ, USA) and a developing solution of toluene-ethyl acetate-acetic acid (60:30:4, *v*/*v*/*v*).

For the determination of the morphological characters, each fungus was inoculated on PDA medium and cultured for 4 days. The colony was observed using a VHX digital microscope equipped with a VH-Z25 lens (Keyence, Osaka, Japan).

### 5.8. Identification of the Fungi

For the identification of each isolated fungus, we collected the fungal mycelia on the GY(2-0.5) agar plate medium by using a pair of forceps, and then disrupted them with zirconium beads using Tris-EDTA-saturated phenol with FastPrep FP100A (Q-BIO 101, Thermo Fisher Scientific, Waltham, MA, USA). After centrifugation, the upper aqueous layer containing DNA was diluted with sterile water and then applied for PCR. The calmodulin (*cmd*) gene of FUT-A1 was amplified by PCR using the enzyme KOD-Plus (Toyobo, Osaka, Japan) and CMDF1 and CMDR1 primers [52]. The resulting PCR product was sequenced. The sequence data were then compared with those of the DNA Data Bank of Japan (DDBJ) by performing a BLAST search. The genomic nucleotide sequence data for the *cmd* gene of the fungi obtained were deposited in the DDBJ/EMBL/GenBank nucleotide sequence database under accession no. LC431242.
